# Predictors of esophageal candidiasis among patients attending endoscopy unit in a tertiary hospital, Tanzania: a retrospective cross-sectional study

**DOI:** 10.4314/ahs.v18i1.10

**Published:** 2018-03

**Authors:** Martha F Mushi, Nathaniel Ngeta, Mariam M Mirambo, Stephen E Mshana

**Affiliations:** 1 Microbiology and Immunology Department; Weill Bugando School of Medicine, P.O. Box 1464, Mwanza, Tanzania; 2 Department of Internal Medicine Weill Bugando School of Medicine. P.O. Box 1464 Mwanza, Tanzania

**Keywords:** Esophagogastroduodenoscopy, esophageal candidiasis

## Abstract

**Background:**

Esophageal candidiasis is a common disease among patients with impaired cell mediated immunity. In the current study, we report esophageal candidiasis among patients with various co-morbidities attending the endoscopic unit at the Bugando Medical Centre.

**Methods:**

This retrospective study was conducted from June to September 2015. All data of the patients who attended the endoscopic unit between 2009 and 2014 were retrieved and analyzed.

**Results:**

A total of 622 patients who underwent oesophagogastroduodenoscopy were analyzed. A slight majority 334/622(53.7%) of patients were female. Out of 622 patients; 35(5.6%) had esophageal candidiasis. Decrease in age (OR 1.1, 95%CI; 1.0–1.1), female sex (OR 3.8, 95%CI; 1.1–13.1), drinking alcohol (OR 17.1, 95%CI; 4.9–58.9), smoking (OR 8.3, 95%CI; 1.7–41.0), antibiotic use (OR 5.7, 95%CI; 2.0–16.4), positive HIV status (OR 10.3, 95%CI; 4.6–6.0) and presence of peptic ulcer disease (OR 13.2, 95%CI; 3.5–49.0) independently predicted esophageal candidiasis.

**Conclusion:**

Patients with a history of drinking alcohol, smoking, use of antibiotics and those with chronic diseases such as peptic ulcers were at high risk of developing esophageal candidiasis. Further studies are needed to identify *Candida spp.* and their anti-fungal susceptibility for proper management of esophageal candidiasis in HIV and non-HIV individuals.

## Background

Esophageal candidiasis (EC) is one of the major opportunistic fungal infections that occur in over 90% of the HIV infected individuals during the course of their illness^1^,^2^. Esophageal candidiasis has also been reported to occur in 39.1% of patients receiving anti-cancer treatment^3^. In HIV negative population, co-morbidities have been found to increase the risk of developing EC^4^–^7^. Other factors that increase the risk of EC include intensive chemotherapy, chronic use of corticosteroids, antibiotic therapy and alcoholism^8^,^9^.

In low-income countries, the diagnosis of EC mainly depends on the clinical presentations; EC commonly presents with dysphagia, retrosternal chest pain and a feeling of obstruction in the esophagus upon swallowing. It is estimated that 68.6% of patients with EC present with dysphagia^10^. In high-income countries, brushing technique following oesophagogastroduodenoscopy (OGD) then culture and sensitivity is routinely performed for the diagnoses of EC while in low-income countries this practice is not routinely done^11^. Many studies in low-income countries have focused on esophageal candidiasis among HIV infected population. This study investigated the magnitude and factors associated with EC among patients attending the endoscopy unit. In addition, the cases of EC among non-HIV infected population are presented in detail.

## Methodology

### Study design and period

This retrospective cross-sectional study was conducted at the endoscopy unit of the Bugando Medical Centre (BMC) a tertiary hospital in Mwanza, Tanzania. BMC endoscopic unit performs a minimum of 80 endoscopic procedures per month. It provides endoscopic services for approximately 13 million people from eight regions in the lake zone of the country. The study was conducted from June 2015 to September 2015. Data from all patients attending the endoscopy unit between 19^th^ August 2009 and 19^th^ September 2014 were extracted and analyzed.

### Data collection and study variables

Checklist was used to collect patients' information from hospital files and the endoscopy unit log book. Independent variables such as socio-demographic characteristics (age, sex, marital status, cigarette smoking and alcohol use) and clinical data (corticosteroid use, antibiotic use, HIV sero-status, diabetic and history of peptic ulcer diseases) were extracted. In this study the diagnosis of esophageal candidiasis was made during oesophagogastroduodenoscopy and was defined by the presence of white or slightly yellowish plaque like lesions on the esophageal mucosa as described in a previous study^12^.

### Ethical consideration

Ethical clearance to conduct this study was granted by the Joint CUHAS/BMC research ethics and review committee (CREC) with certificate number CREC/045/2014. Permission to conduct the study was sought from the endoscopy unit and all data was kept confidential.

### Data analysis

Data were entered and cleaned using Microsoft Excel software and analyzed using STATA Version 11. Age was summarized using mean ± standard deviations. Sex, alcohol use, smoking, antibiotic use, HIV status and presence of peptic ulcer diseases (PUD) were summarized as proportions. Univariate and multivariate logistic regression analysis was done to determine factors associated with esophageal candidiasis among patients attending endoscopy unit. All factors from the literature known to predict EC were subjected to multivariate analysis. A p value of less than 0.05 was considered as statistically significant at 95% confidence interval.

## Results

### Demographic characteristics

Of the 625 patients who attended the endoscopy unit, 622 patients were enrolled in this study. Three patients were excluded because they had no data on clinical presentations or indication for the endoscopy. The mean age of enrolled patients was 41.59 +15.38 years. A slightmajority of patients were female 334(53.7%). A total of 396(63.7%) participants were unemployed and 121(19.5%) had a history of antibiotic use. Most of patients were HIV negative 554(89.07%) as shown in table 1. The co-morbidities among the studied population included peptic ulcer diseases 85(13.67), diabetics 14(2.25), cirrhosis 14(2.25), asthma 5(0.80) and cancer 4(0.64).

**Table 1:** Baseline characteristics of the 622 studied participants

Variable	Frequency	Percentage
Age*	41.5±15.38	

**Sex**		

Male	288	46.3%
Female	334	53.7%

**Occupation**		

Employed	226	39.3
Unemployed	396	63.7

**Alcohol use**		

No	524	84.2
Yes	98	15.8

**Smoking**		

No	581	93.4
Yes	41	6.6

**Clinical presentation**		

Epi. Pain	315	50.6
Upper GI bleeding	233	37.5
Dysphagia	55	8.8

**Corticosteroid use**		

No	618	99.4
Yes	4	0.6

**Antibiotics use**		

No	501	80.5
Yes	121	19.5

**HIV**		

Negative	554	89.1
Positive	15	2.4
Unknown	53	8.5

**Diabetic**		

No	608	97.7
Yes	14	2.3

**PUD**		

No	537	86.3
Yes	85	13.7

Note; Ep. Pain : epigastric pain, UGIB: upper gastro intestinal bleeding and PUD : peptic ulcer diseases,

* Mean has been reported

### Clinical presentation

Of 622 participants, 315(50.6%) presented at endoscopy unit with epigastric pain/abdominal pain, 19(3.05%) with dyspepsia, 55(8.84%) presented with dysphagia and 233 (37.5%) presented with upper gastro-intestinal bleeding.

### Prevalence and predictors of esophageal candidiasis

Out 622 patients, 35 (5.63%) were diagnosed to have esophageal candidiasis. By multivariate logistic regression analysis: decrease in age OR 1.11, 95% CI; 1.02–1.12; p=0.007, sex(OR 3.8, 95% CI; 1.11–13.13; p=0.033), alcohol use(OR 17.1, 95%CI; 4.94–58.9; p<0.001), smoking (OR 8.3, 95%CI; 1.68–41; p=0.009), antibiotic use (OR 5.74, 95% CI; 2–16.4; p<0.001), positive HIV status (OR 10.3, 95%CI; 2.27–45; p<0.001) and having peptic ulcer diseases (OR 13.2, 95%CI; 3.53–49; p<0.001) independently predicted EC as shown in table 2.

**Table 2:** Factors associated with esophageal candidiasis

Variable	Univariate			Multivariate	
	EC n (%)	OR(95%CI)	P value	OR(95%CI)	P
Age*	39.4±15.1	1.02(0.99–1.04)	0.386	1.11(1.02–1.12)	0.01
**Sex**					
Male (288)	14(4.2)	1			
Female (334)	21(7.3)	1.79(0.89–3.6)	0.0098	3.8(1.11–13.13)	0.033
**Alcohol**					
No (524)	14(2.7)	1			
Yes (98)	21(21.43)	9.93(4.8–20)	<0.001	17.1(4.94–58.87)	<0.001
**Smoking**					
No (581)	27(4.7)	1			
Yes(41)	8(19.51)	4.97(2.09–11.79)	<0.001	8.3(1.68–41)	0.009
**Antibiotics use**					
No (501)	17(3.39)	1			
Yes (121)	18(14.88)	4.97(2.48–9.98)	<0.001	5.74(2–16.43)	<0.001
**HIV**					
Negative (554)	20(3.61)	1			
Positive (15)	8(53.33)	30.51(10.07–92.42)	<0.001		
Unknown (53)	7(13.21)	4.06(1.63–10.11)	0.003	10.34(2.27–45)	<0.001
**PUD**					
No (537)	27(5.03)	1			
Yes (85)	8(9.41)	1.96(0.86–4.47)	0.109	13.17(3.53–49.09)	<0.001

Note: PUD: peptic ulcer diseases

### Esophageal candidiasis among HIV negative patients

Of 554 HIV negative patients, 20(3.6%) had EC. The common clinical presentation of the HIV negative patients with EC was epigastric pain 70% (14/20). Majority of HIV negative patients with EC were using alcohol 70%(14/20) and had a history of using antibiotics prior to endoscopic examinations 55% (11/20) as shown in table 3. Common co-morbidities among 20 HIV negative patients with EC were peptic ulcer disease 7(35%) and diabetes mellitus 6(30%). Of the 20 HIV negative patients with EC, 4 (20%) did not have any co-morbidity but reported taking alcohol (table 3).

**Table 3:** Characteristics of HIV negative patients with EC

S/number	Sex	Age	Presentation	Alcohol	Smoking	Antibiotic	Diabetic	Cirrhosis	PUD	Asthma
1	Male	20	No	No	No	No	No	No	Yes	No
7	Male	40	Ep. pain	Yes	No	Yes	No	No	Yes	
8	Female	38	Ep. pain	Yes	No	No	Yes	No	No	No
9	Female	47	Ep. pain	No	No	No	Yes	No	No	No
10	Male	77	Ep. pain	Yes	Yes	Yes	No	No	No	No
11	Female	38	Ep. pain	No	No	Yes	No	No	Yes	No
12	Male	34	UGIB	Yes	Yes	No	No	No	Yes	No
13	Female	60	Ep. pain	Yes	No	Yes	No	No	Yes	No
14	Male	28	Ep. pain	Yes	Yes	No	No	No	No	No
15	Male	40	Dysphagia	Yes	No	No	Yes	No	Yes	No
16	Male	36	Ep. pain	Yes	No	Yes	Yes	No	No	No
17	Male	44	UGIB	Yes	Yes	No	No	Yes	No	No
18	Female	62	Ep. pain	No	No	No	Yes	No	No	No
19	Female	33	Ep. pain	Yes	No	Yes	No	No	Yes	No
20	Male	23	Ep. pain	No	No	Yes	No	No	No	Yes

Note; Ep. Pain is epigastric pain and UGIB is upper gastro intestinal bleeding

## Discussion

Esophageal candidiasis is the commonest cause of esophagitis among HIV infected individual. As documented in previous studies^2^,^13^, half of the patients in the present study had epigastric pain (50.6%) This can be explained by the possibility that *Candida spp.* might have caused gastritis^14^. Other documented complaints include odynophagia, dysphagia and retrosternal pain^2^,^15^.

With improvement of the endoscopic services, EC has been reported in HIV negative patients^13^,^16^, as evidenced in the current study whereby 3.6% of HIV negative patients had EC. In HIV negative patients, the EC is normally associated with peptic ulcer diseases, diabetes and prolonged use of antibiotics^4^, these factors were confirmed in the current study.

In diabetic patients, EC is linked with depressed immune system and the stasis of the esophageal content^17^. Esophageal candidiasis among patients with PUD is linked to the chronic use of the proton pump inhibitors which lead to hypochlorhydria which in turn alters the stomach bacteria flora colonization and increases the risk of candida over growth^10^,^18^.

Use of broad spectrum antibiotics results in normal flora disturbances leading to over growth of *Candida spp.* which might progress to EC^4^,^10^. In the present study patients with a history of antibiotic use had 14 times odds of getting esophageal candidiasis than patients with no history of antibiotic use. As it was described in other studies^18^,^19^, alcohol drinking and smoking were confirmed in this study as predictors of getting EC. This can be explained by the fact that alcohol has the effect of elevating the gastric pH and augmenting the colonization of the esophagus by oral cavity bacteria and yeast^19^.

Despite the significance of the data presented in this study some limitations have been noted; firstly, the study was retrospective; hence the risk factors can be under estimated, secondly the sensitivity and specificity of the clinical diagnosis of EC by endoscopy might have affected the outcome.

## Conclusion

Patients with history of drinking alcohol, smoking, use of antibiotics and those with chronic diseases such as peptic ulcers are at high odds of developing esophageal candidiasis. Further studies to identify *Candida spp.* and their anti-fungal susceptibility are recommended for proper management of esophageal candidiasis in HIV and non-HIV individuals.
